# Hyperandrogenism and menstrual imbalance are the best predictors of metformin response in PCOS patients

**DOI:** 10.1186/s12958-021-00876-0

**Published:** 2022-01-04

**Authors:** Emanuele Garzia, Valentina Galiano, Giovanni Marfia, Stefania Navone, Enzo Grossi, Anna Maria Marconi

**Affiliations:** 1 Reproductive Medicine Unit, Department of Mother and Child, San Paolo Hospital Medical School, ASST Santi Paolo e Carlo, 20142 via di Rudinì, 8 –, Milano, Italy; 2 Istituto di Medicina Aerospaziale “A. Mosso”, Aeronautica Militare, Milano, Italy; 3 Laboratory of Experimental Neurosurgery and Cell Therapy, Neurosurgery Unit, Fondazione IRCCS Ca’ Granda Ospedale Maggiore Policlinico, Milano, Italy; 4 ”Aldo Ravelli” Research Center, Milano, Italy; 5 Villa Santa Maria Foundation, Tavernerio, Italy; 6 Gynecology and Obstetrics Unit, Department of Mother and Child, San Paolo Hospital Medical School, Department of Health Sciences, University of Milano, Milano, Italy

**Keywords:** PCOS, Insulin resistance, Hyperandrogenism, Metformin, Oligo-amenorrhea

## Abstract

**Background:**

Moving from the correlation between insulin-resistance and PCOS, metformin has been administered in some PCOS women improving ovulatory and metabolic functions and decreasing androgen levels. Inconsistency and unpredictability of response to metformin limit its extensive use. Aim of this study was to identify reliable predictors of response to metformin therapy for weight loss and reduction in plasma androgen levels using ANNs (artificial neural networks).

**Methods:**

One hundred eight consecutive women with PCOS (ESHRE/ASRM 2003 Rotterdam criteria) treated with metformin 1500 mg/day, at inclusion and every 6 months underwent to a complete clinical, endocrine/metabolic assessment and ultrasonographic evaluation. Therapy outcomes were BMI reduction (≥1 kg/m^2^) in overweight/obese and free-androgen-index (FAI) decrease (≥1%) in hyperandrogenemic women. Semantic connectivity maps (SCMs) were obtained through Auto-CM, a fourth generation ANN, to compare patients’ baseline clinical features to the treatment outcomes. Multivariate logistic regression analysis was used to assess the major predictor in drop-out patients and the associated risk.

**Results:**

At 6 months 54 out of 103 (52,4%) obese patients showed BMI reduction and 45 out of 89 (50,6%) hyperandrogenemic women showed FAI decrease. The further response rates at 12 months were 30,6 and 47%, respectively. SCMs showed a clear polarization for both the outcomes with elevated accuracy. Treatment responsiveness resulted strictly related to oligo-amenorrhea and hyperandrogenemia at baseline. In addition, lower serum testosterone levels at baseline were found to be the major predictor of treatment discontinuation.

**Conclusions:**

In women with PCOS, menstrual pattern imbalance and ovarian androgens excess are the best predictors of metformin response. They may pave the way for a rethinking of the criteria for evaluating hyperandrogenism in order to better define the large population included in the diagnosis of PCOS. Baseline plasma testosterone level can serve as a sensitive marker to predict treatment compliance.

**Supplementary Information:**

The online version contains supplementary material available at 10.1186/s12958-021-00876-0.

## Background

Polycystic ovary syndrome (PCOS) is the most common endocrine disorder in women of reproductive age. Its etiology is complex and poorly understood. PCOS is clinically characterized by hyperandrogenism and ovulatory disturbances frequently associated with metabolic features such as insulin resistance, obesity and dyslipidemia [1]. With the aim of correctly classifying PCOS, three sets of diagnostic criteria have been proposed: all three definitions include hyperandrogenism, either clinical or biochemical and oligo-anovulation with the Rotterdam criteria firstly involving the polycystic ovarian morphology on ultrasound (PCOM) [2–4]. In recent decades, the body of evidence indicating that insulin resistance plays a key role in the pathogenesis of PCOS has grown significantly. Hyperinsulinemia aggravates the hormonal and ovulatory dysfunctions [5] by inducing an excessive production of androgens and a decrease in serum sex hormone binding globulin (SHBG). The insulin-induced increase in androgens is primarily due to the direct effect on the steroidogenesis of the ovarian theca cells [6] and also to the inhibition of insulin-like growth factor 1 (IGF-1) binding protein production by the liver with a subsequent increase in free IGF-1 [7]. Insulin resistance affects 65–70% of women with PCOS [8]: it is more common in overweight, but it can also occur in normal weight women [9, 10]. The treatment of PCOS, given its complexity, is not unique. While on the one hand a symptomatic approach may be appropriate for some women, in subjects with severe endocrine and metabolic abnormalities, pathogenesis-focused treatment is required to act on the various clinical manifestations and to allow long-lasting effects [11]. Since the introduction of metformin as a therapeutic option in PCOS in the 1990s [12], many studies have suggested that, through the reduction of insulin resistance, it improves metabolic alterations, decreases androgen levels and improves menstrual and ovulatory function [13, 14], so much so that several authors currently recommend the use of metformin in PCOS women with IGT or hyperinsulinemic [11]. However, the extensive use of metformin in women with PCOS is limited by the variability in treatment response, as up to 30% of women are “non responders” [15], and by the rate of withdrawals, principally due to gastrointestinal side effects and inability to comply [16]. To date, despite efforts, there are no reliable parameters that can accurately predict individual response to metformin treatment [17, 18].

The artificial neural networks (ANNs) are adaptive systems inspired by the functioning processes of the human brain [19]. ANNs are able to modify their internal structure in relation to a functional target and are particularly suited for solving nonlinear problems. While conventional statistical methods, such as logistic regression or discriminant analysis, require a limited number of independent variables, ANNs may use all information provided by the variables and are nowise affected by the nonlinear correlation between independent variables [20–22]. Therefore, in a heterogeneous condition such as PCOS, this mathematical approach can be particularly useful.

The aim of the present study was to identify reliable predictors of response to metformin in women with PCOS using the ANNs. To evaluate the effectiveness of metformin treatment, we chose two parameters that could be simply measured and compared: the reduction in body weight and plasmatic androgens. As secondary outcomes, we evaluated the effects of metformin on menstrual function, hirsutism, body fat distribution and glyco-lipidic profiles.

## Methods

### Subjects

One hundred and eight consecutive women with PCOS, referred to the Gynecological Endocrinology outpatient clinic of the Reproductive Medicine Unit of the San Paolo Hospital Medical School, were enrolled in this longitudinal study. Exclusion criteria were the use of oral contraceptives, other medications capable to affect the hypothalamic–pituitary–ovarian axis, chemotherapy or immunosuppressive agents in the previous 6 months or the presence of any concomitant systemic disease including diabetes, dyslipidemia and hypertension. The diagnosis of PCOS was based on the ESHRE/ASRM Rotterdam consensus criteria [3], which requires at least two of the following: oligomenorrhea/amenorrhea; clinical/biochemical hyperandrogenism; PCOM. Oligomenorrhea was defined as ≤3 spontaneous menstrual cycles in 6 months. Hyperandrogenemia was defined as free androgen index (FAI) ≥6%, calculated as total Testosterone (ng/mL) × 347/sex hormone binding globulin (SHBG) (nmol/L) [23, 24]; Δ4-androstenedione (A) ≥3.9 ng/mL or dehydroepiandrosterone sulfate (DHEAS) ≥410 μg/dL. Clinical hyperandrogenism was assessed by the presence of persistent acne [25], hirsutism (modified Ferriman–Gallwey score ≥ 8) [26, 27] or androgenic alopecia [28]. Other causes of hyperandrogenism and anovulation, namely thyroid dysfunctions, hyperprolactinemia, hypercortisolism, congenital adrenal hyperplasia and androgen-secreting tumors were excluded. The ultrasound assessment of PCOM was performed with endovaginal ultrasound transducers (Esaote myLab X6) with a frequency bandwidth of 8 MHz in presence of an antral follicle count (AFC) [29] per ovary of ≥20 and/or an ovarian volume ≥ 10 ml was present, ensuring no corpora lutea, cysts or dominant follicles were present [30]. Body mass index (BMI) was calculated as weight in kg/(height in m)^2^. Waist and hip circumferences were measured to the nearest centimeter with a soft tape at the narrowest part of the torso and at the widest part of the gluteal region and the waist/hip ratio calculated (WHR) [31–33]. The homeostatic model assessment of insulin resistance (HOMA-IR) was calculated as [glucose (mg/dL) × 0.05551] x insulin (IU/mL)/22.5 [34]. The Institutional Review Board of the San Paolo Hospital Medical School approved the treatment protocol and signed informed consents were obtained from all patients before commencing the data collection.

### Protocol

At inclusion (m0) all women underwent a complete clinical evaluation, an endocrine and metabolic laboratory assessment and a transvaginal ultrasound. Overnight fasting blood samples were collected in amenorrhoeic women after medroxyprogesterone induced (Farlutal 10 mg Pfizer Italia s.r.l.) withdrawal bleeding, and in the early follicular phase in women with spontaneous menses, to assess blood glucose, insulin, gonadotropins, 17β estradiol (E2), total testosterone (T), DHEAS, A, SHBG, triglycerides, total and fractionated cholesterol. Assay methods, intra- and inter-assay coefficients of variation are reported in the Supplementary Data. Women were then prescribed metformin 500 mg/d (Metforal 500 mg Laboratori Guidotti SPA) in the first week, progressively increased to 1500 mg/d in 2 weeks. In women reporting side effects, a dose reduction to 1000 mg/d was allowed for a maximum of 2 weeks, while in case of intolerable side effects it was advised to stop treatment. The women were asked not to change their eating habits and to report the usual diet in a periodic questionnaire. They were also instructed to record in a diary the menstrual bleedings. After 6 (m6) and 12 (m12) months of treatment, women underwent the same complete assessment as m0. We considered response to treatment the reduction in body weight and plasmatic androgens as well as the improvement in menstrual function, hirsutism, body fat distribution and glyco-lipidic profiles. In consideration that dropout might identify a subpopulation of women less responsive to drug treatment, we compared this group with that of women treated up to m12.

The primary outcomes were the reduction of BMI (Δ ≥ 1 kg/m^2^) in overweight and obese women (*n* = 103) and the decrease of FAI (Δ ≥ 1%) in hyperandrogenemic (considered as FAI ≥6%) women (*n* = 89).

### Statistical analysis

Data are presented as mean ± standard deviation. Discrete variables were reported as counts or percentages. Recorded parameters were tested for normality using the Shapiro-Wilk test, and when no-normally distributed, differences between parameters at baseline and after six and 12 months of therapy, were calculated by nonparametric Friedman’s two-way rank analysis of variance with correlated samples. Nonparametric Mann-Whitney test was performed to compare the parameters of the drop out subpopulation and the women who accomplished 12 months of therapy. Logistic regression analysis was used to assess the predictive value of variables for drop-out events and the relative risk of drop-out in the study. Statistical analysis was carried out using IBM SPSS Statistics 26.0 software. Data acquisition was performed blindly. The tests were considered statistically significant when *p* < 0.05.

### Artificial neural networks

We employed a stepwise approach consisting in a predictivity modelling followed by data mining. The predictivity approach aimed to establish the optimal variables mix to distinguish responders to metformin with regard a critical reduction of BMI and FAI**.** It was realized through TWIST, an evolutionary algorithm based on a seminal paper about Genetic Doping Systems [35], used in bio medicine from 2003 with many peer review applicative papers [36]. The procedure involves the applied the TWIST (Training with Input Selection and Testing) system, an iterative hybrid ML system coupling an evolutionary algorithm named Gen-D and a backpropagation neural network, to subdivide the data set into training and testing sets as well as to select features yielding the maximum amount of information. As described in previous studies, the TWIST algorithm is a complex algorithm able to search for the best distribution of the global data set divided into 2 optimally balanced subsets containing a minimum number of input features useful for optimal pattern recognition. At the end of TWIST iterations, a robust set of features were selected to be used as input for artificial neural networks. Back Propagation ANN were used to develop a predictive model to distinguish subjects belonging to the 2 diagnostic classes (responders vs non responders). Models’ performances were tested with training/testing cross-validation procedures. Data mining has been carried out with Auto-CM, a fourth-generation ANN developed at Semeion Research Centre – Italy. Auto-CM is able to compute and graph a “semantic connectivity map” (SCM) which (i) preserves nonlinear associations among variables, (ii) captures elusive connection schemes among clusters, and (iii) highlights complex similarities among variables. The 3-layers architecture and the mathematical models of Auto-CM have been described elsewhere [37]. This model has both a training and a learning phase. After the former, Auto-CM determines the “weights” of the vectors matrix, which (i) represent the warped landscape of the dataset and (ii) permit a direct interpretation. Indeed, these weights are proportional to the strength of many-to-many associations across all variables and can be easily visualized by transforming them into physical distances: variables whose connection weights are higher get relatively closer and vice versa. By applying a mathematical filter (i.e. Minimum Spanning Tree) [38] to the matrix of distances it generates the SCM. To obtain the best performance from the Auto-CM analysis it was necessary to transform the continuous variables contained in the database into nominal variables. The data have been dichotomized, before the application of auto-CM system, using the medians as cut-off. Since the values of the baseline parameters are continuous variables, we named every feature as high or low if it was identifying the data above or below the median (Table 1).

**Table 1:** Clinical, ultrasonographic, biochemical parameters of the enrolled women at baseline (m0) and at the 6 (m6) and 12 (m12) months follow up following metformin (1500 mg/d) treatment

***Parameter***	*m0 (n = 108)*	*median*	*m6 (n = 82)*	*P (m6:m0)*	*m12 (n = 53)*	*P (m12:m6)*
**Age (years)**	28.38 ± 5.53					
**Weight (Kg)**	87.2 ± 18.1	84	82.2 ± 18.1	**0.0001**	80.05 ± 16.9	**0.005**
**BMI (Kg/m**^**2**^**)**	32.8 ± 5.9	32	30.7 ± 5.9	**0.0001**	30.19 ± 5.7	**0.002**
**Glucose (mg/dL)**	91.64 ± 9.66	90	87.90 ± 9.57	**0.026**	85.91 ± 8.84	0.569
**Insulin (μU/mL)**	21.03 ± 11.6	18	15.8 ± 8.5	**0.001**	13.8 ± 7.5	**0.02**
**HOMA-IR**	4.83 ± 2.74	4	3.45 ± 1.95	**0.0001**	2.96 ± 1.71	**0.035**
**Triglycerides (mg/dL)**	121.6 ± 61.9	110	121.6 ± 66.4	0.06	112.8 ± 58.5	0.425
**Total cholesterol (mg/dL)**	189.9 ± 32.5	185	188.6 ± 32.3	0.06	184.1 ± 35.2	0.856
**LDL cholesterol (mg/dL)**	120.8 ± 26.8	120	115.3 ± 28.2	**0.008**	118.1 ± 29.0	0.620
**HDL cholesterol (mg/dL)**	44.1 ± 8.9	43	45.7 ± 9.5	**0.003**	45.6 ± 9.5	0.674
**WHR**	0.91 ± 0.1	0,91	0.89 ± 0.09	0.067	0.88 ± 0.09	0.067
**T (ng/mL)**	0.70 ± 0.31	0,60	0.60 ± 0.29	**0.001**	0.53 ± 0.24	**0.027**
**SHBG (nmol/L)**	23.1 ± 10.4	22	26.4 ± 11.7	**0.0001**	29.7 ± 13.6	**0.002**
**FAI %**	13.2 ± 7.01	9	12.1 ± 6.6	**0.0001**	11.9 ± 6.04	**0.0001**
**DHEAS (μg/dL)**	291.7 ± 125.4	270	278.8 ± 123.2	0.184	272.8 ± 99.8	0.366
**Δ4A (ng/mL)**	4.02 ± 0.65	4	3.89 ± 0.69	0.058	3.85 ± 0.62	0.175
**F-G**	12.5 ± 6.9	12	12.3 ± 6.5	**0.05**	11.9 ± 6.04	**0.008**
**LH/FSH**	1.44 ± 0.9	1,25	1.19 ± 0.65	**0.169**	1.09 ± 0.53	**0.327**
**Menses/6 months**	2.0 ± 1.7	2	3.5 ± 2.2	**0.0001**	3.6 ± 2.2	**0.024**
**PCOM (US)**	73%		52%		63%	
**AFC (num- bothovaries)**	41 ± 9	40	39 ± 7	**0.0001**	38 ± 6	**0.040**

*BMI* body mass index; *HOMA-IR* homeostatic model assessment of insulin resistance; *WHR* waist to hip ratio; *T* total testosterone; *SHBG* sex hormone binding globulin; *FAI* free-androgen-index; *DHEAS* dehydroepiandrosterone sulfate; *Δ4A* Δ4-androstenedione; *F-G* Ferriman–Gallwey score; *LH* luteinizing hormone; *FSH* follicle stimulating hormone; *PCOM* polycystic ovarian morphology; *AFC* antral follicle count

## Results

**Figure 1:**
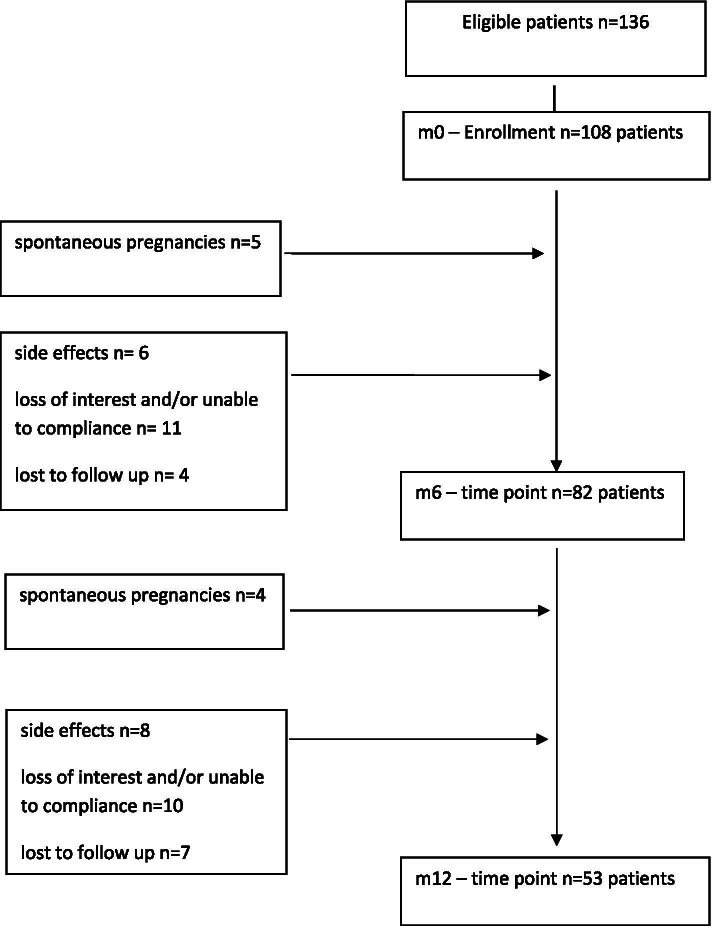
Flow chart of the study population

Of 136 potentially eligible women 108 (79.4%) met the inclusion criteria, gave the consent and started the metformin treatment. 75.9% of the enrolled population (82 women) properly took the medication for 6 months while 21 women dropped out of therapy due to intolerable side effects (*n* = 6), poor advantage perception (*n* = 11) or were lost to follow up (*n* = 4). About 5% (5 women) became pregnant and were excluded from the study. From m6 to m12, another 25 women discontinued treatment: 8 due to side effects, 10 due to loss of interest and 7 lost to follow up, while 4 women became pregnant. Fifty-three women continuously received metformin and were assessed at 12 months (m12) (Fig. 1). The anthropometric, endocrine and metabolic measures of the enrolled women at m0, m6 and m12 are shown in Table 1.

**Figure 2:**
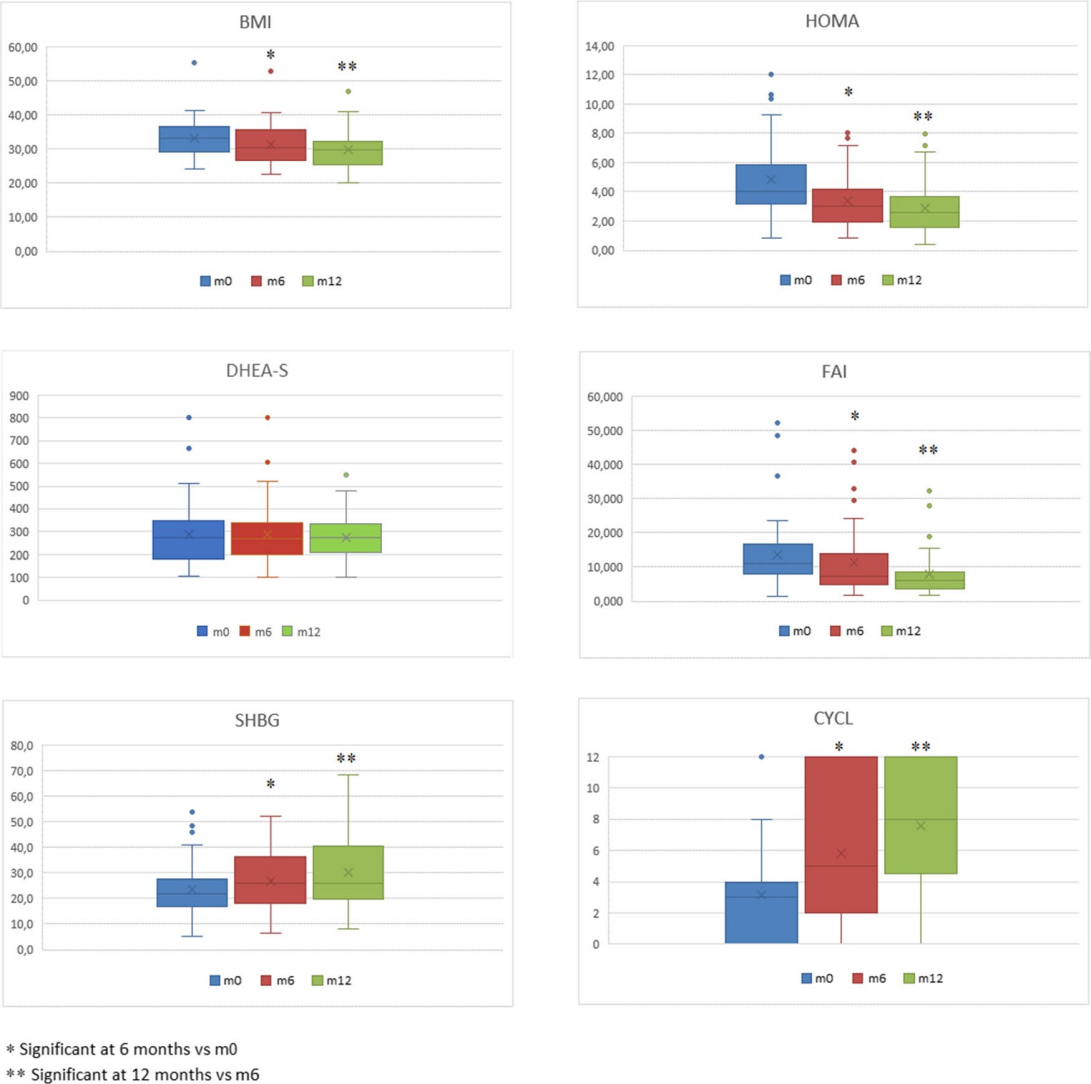
The measurements of BMI, HOMA-IR, FAI, SHBG, DHEA-S and menstrual regularity at baseline, at m6 and m12 timepoints in the subset of women who accomplished 12 months of metformin therapy (53 patients).

The evaluation of dietary ratios did not reveal substantial differences in caloric intake between women and in the same subjects over time. Between m0 and m6 fasting glucose, insulin, LDL cholesterol and T levels decreased significantly as did BMI, Ferriman-Gallwey score, LH/FSH ratio and AFC, whereas the number of menses per 6 months, plasma SHBG and HDL cholesterol increased. At m12, compared to m6, all these values, except the lipid profile, are further improved. Regarding androgens, both treatment periods resulted in reduced T levels, increased SHBG with a consequent effect on FAI, in the absence of significant changes in the levels of A and DHEAS. (Table 1, Fig. 2).

Fifty-four (52.4%) out of 103 overweight/obese women reduced BMI ≥1 kg/m2 whereas 45 (50.6%) out of 89 hyperandrogenemic women reduced FAI ≥1%. At m12 we observed a further improvement in BMI in 22 (30.6%) out of 72 women and in FAI in 24 (47.1%) out of 51, respectively

**Figure 3:**
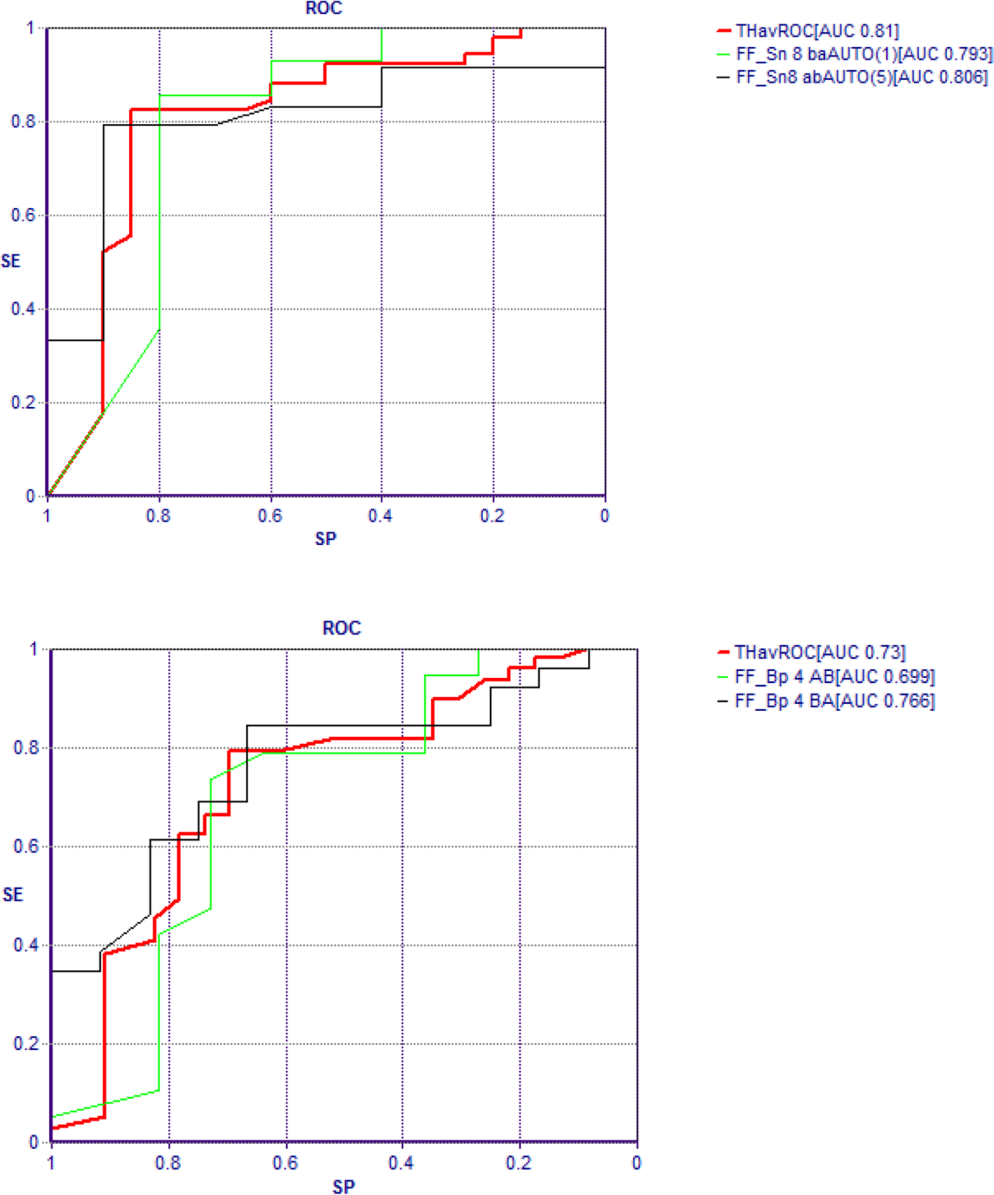
The ROC AUC curves obtained with the application of machine learning systems (Feed forward Sine-Net artificial neural network algorithm FF_Sn with 8 hidden units) and back propagation algorithm (FF_BP with 4 hidden units). Prediction of BMI response after modelling Sine-net ANN on 4 variables (picture above) and prediction of FAI response after modelling with Sine-net ANN on 6 variables (picture below). In red average curve of two experiments: ab and ba training-testing sequence

**Figure 4:**
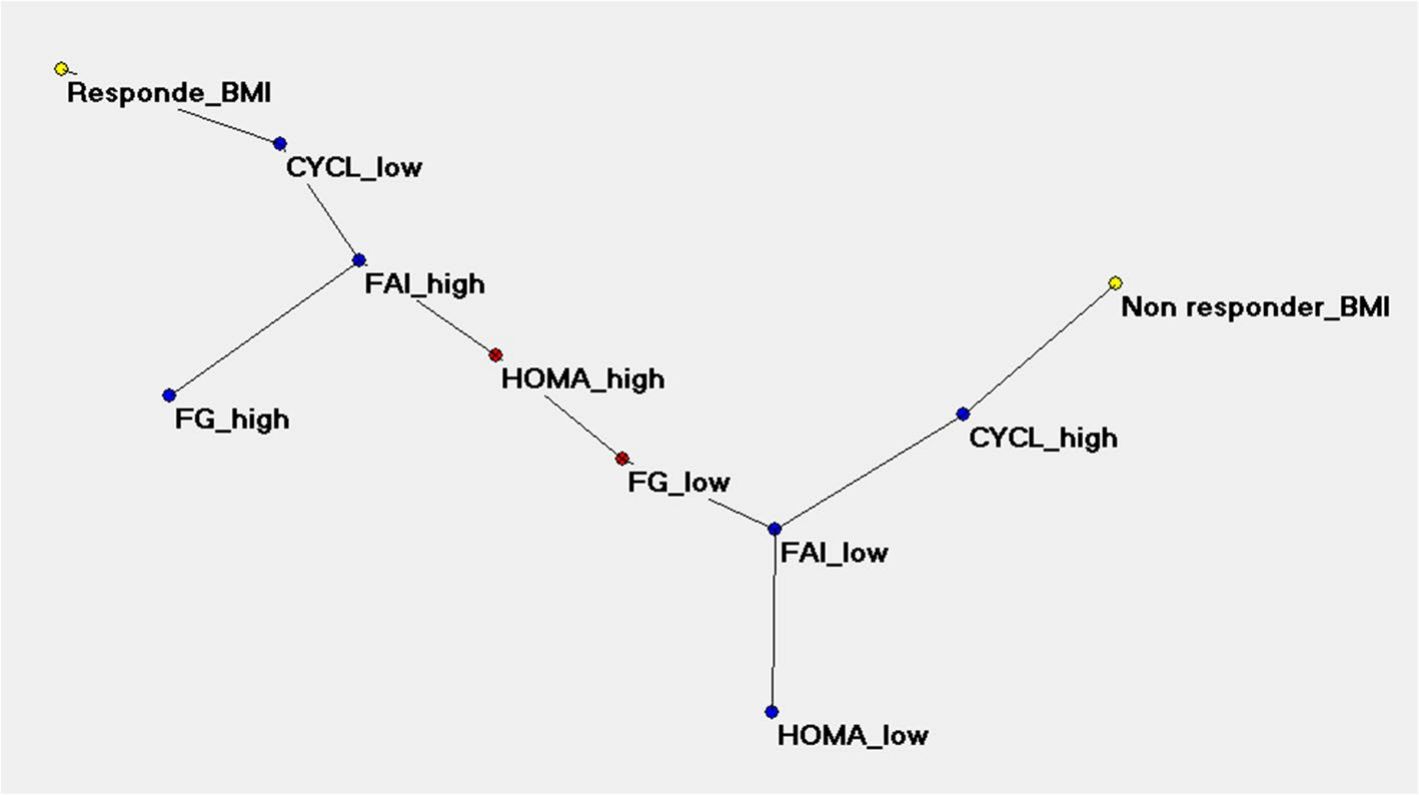
Semantic connectivity map (SCM) of the BMI response. Auto-CM, a fourth generation ANN developed at Semeion Research Centre (Italy), is able to outline the semantic connectivity maps transforming in physical distances the strength of the association among the variables. In order to transform the continuous into nominal variables the data have been dichotomized using the median as cut-off. The metformin responsiveness proved to be related, in order of priority, to oligo-amenorrhea (CYCL LOW: <4 menses/12 months), hyperandrogenemia (FAI HIGH: >9 %), hirsutism (FG HIGH: Ferriman-Gallwey score >12) and insulin-resistance (HOMA HIGH: HOMA-IR >4).

With the ANNs analysis, the TWIST system selected 4 variables connected to BMI response and 6 variables connected to FAI response. The ROC AUC curves obtained with the application of machine learning systems and back propagation algorithm are shown in Fig. 3. The Auto-CM application on the two data sets with high-low transformation produced the SCMs. The “BMI reduction” map was clearly polarized at m6 with 4 variables and 81% accuracy. According to the variables dichotomization, performed using the median value as cut-off, metformin responsiveness was related, in order of priority, to oligo-amenorrhea (CYCL LOW: < 2 menses/6 months), hyperandrogenemia (FAI HIGH: > 9%) and then hirsutism (FG HIGH: Ferriman-Gallwey score > 12) and insulin resistance (HOMA HIGH: HOMA-IR > 4) with a relative priority directly depending by the separation grades from the pole “responder BMI”. Conversely the metformin no responsiveness in body weight reduction was related to CYCL HIGH, FAI LOW, FG LOW and HOMA LOW (Fig. 4).

**Figure 5:**
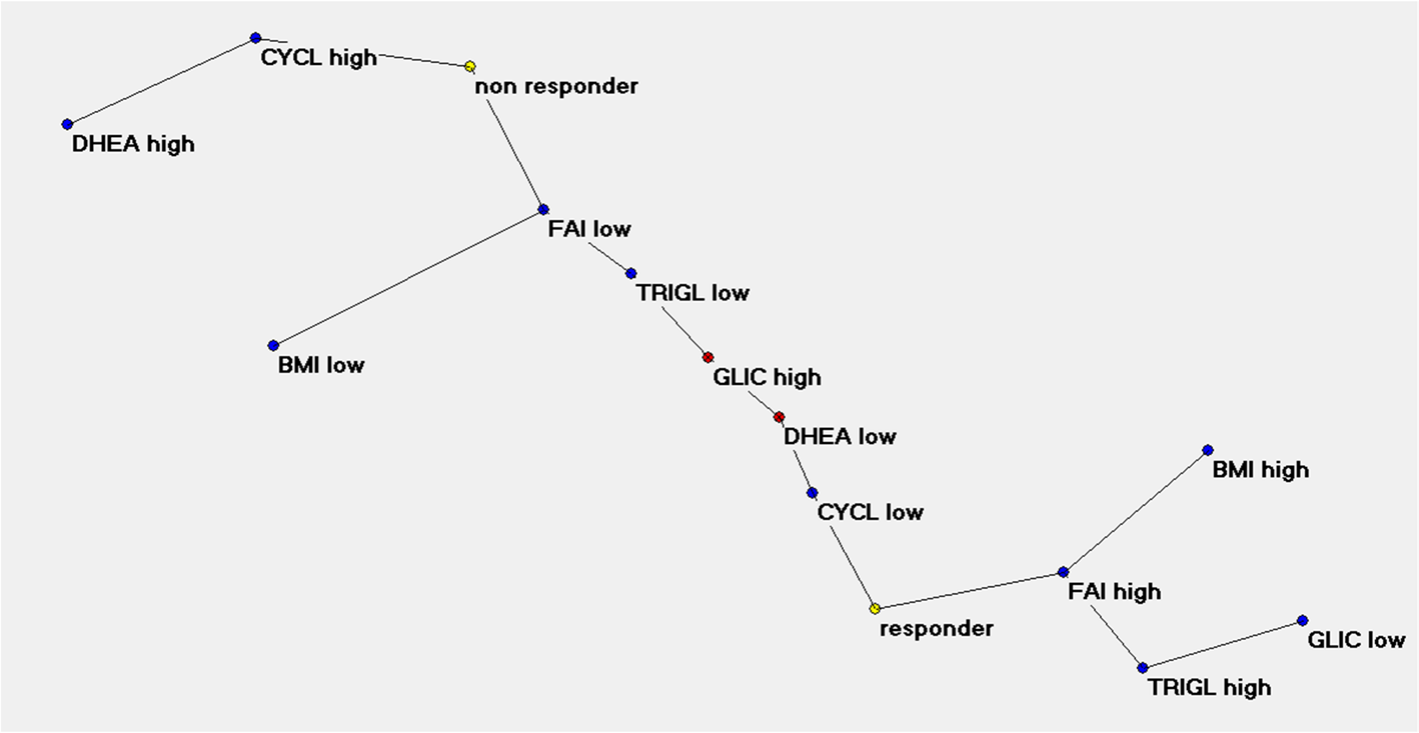
Semantic connectivity map (SCM) of FAI response. The variables related to metformin responsiveness, with a relative priority directly depending by the separation degrees from the pole “responder”, were oligo-amenorrhea (CYCL LOW: <4menses/12months), hyperandrogenemia (FAI HIGH: >9 %), DHEA-S levels (DHEA LOW: <270 μg/dL), body weight (BMI HIGH: >32 kg/m^2^), triglycerides (TRIGL HIGH: >110 mg/dL) and fasting glucose levels (GLIC LOW: <90 mg/dL).

Figure 5 shows the same analysis for “FAI reduction” where, however, the SCM polarized at m12 through 6 variables and 87% accuracy. The variables related to metformin responsiveness were oligo-amenorrhea (CYCL LOW: <2menses/6 months), hyperandrogenemia (FAI HIGH: > 9%), DHEA-S levels (DHEA LOW: < 270 μg/dL), body weight (BMI HIGH: > 32 kg/m^2^), triglycerides (TRIGL HIGH: > 110 mg/dL) and fasting glucose levels (GLIC LOW: < 90 mg/dL).

A substantial proportion of the enrolled population, 46 women, discontinued therapy primarily because of inability to comply or were lost to follow up. Comparing this group with that of women treated up to m12 we found that, at baseline, women who would continue treatment had both significantly higher hyperandrogenism (T and A levels, FAI, FG score) and AFC than those who would discontinue (Table 2). A logistic regression analysis was performed to estimate the major independent factors for women who dropped out of the study during the 12 months of therapy. Interestingly, T was found to be significantly associated with drops-out (Table 3). Specifically, multivariate logistic regression revealed that serum T levels was a significant predictor of drop-out events (*P* = 0.042; odds ratio, 18.723; 95% confidence interval, 1.110–315.880; Table 3). In particular, a negative correlation was found between serum T levels and drop-out events (r = − 0.290, *p* = 0.002) and patients with a serum T levels lower than 0.90 ng/mL had about a 6-fold higher risk to drop-out the study [Exp(B) = 6.557 (CI: 2.044 - 21.036), *p* = 0.002)].

**Table 2:** Baseline clinical, ultrasonographic, biochemical parameters of the population who carried out 12 months of therapy vs the subpopulation who dropped out

	No drop-out (n°82)	Drop-out (n°21)	***P*** value
**Weight (Kg)**	89.1 ± 17.2	84.1 ± 19.1	0.136
**BMI (Kg/m**^**2**^**)**	33.6 ± 5.6	31.6 ± 6.5	0.129
**Glucose (mg/dL)**	91 ± 9	93 ± 9	0.180
**Insulin (μU/mL)**	23 ± 12	19 ± 10	0.096
**HOMA-IR**	5.13 ± 2.89	4.32 ± 2.50	0.143
**Total cholesterol (mg/dL)**	193 ± 32	185 ± 30	0.214
**LDL cholesterol (mg/dL)**	125 ± 27	118 ± 25	0.205
**HDL cholesterol (mg/dL)**	42 ± 7	45 ± 10	0.238
**Triglycerides (mg/dL)**	130 ± 67	111 ± 50	0.188
**WHR**	0.91 ± 0.09	0.89 ± 0.11	0.393
**T (ng/mL)**	0.75 ± 0.35	0.58 ± 0.21	**0.027**
**SHBG (nmol/L)**	22.86 ± 10.28	26.11 ± 12.62	0.257
**FAI %**	13.86 ± 9.76	9.067 ± 5.071	**0.001**
**Δ4A (ng/mL)**	4.11 ± 0.645	3.859 ± 0.624	**0.019**
**DHEAS (μg/dL)**	289 ± 136	301 ± 119	0.321
**F-G**	14.23 ± 6.70	10.57 ± 6.70	**0.007**
**LH/FSH**	1.475 ± 0.725	1.479 ± 1.134	0.230
**Menses/6 months**	1.75 ± 1.63	2.28 ± 1.76	0.082
**PCOM (US)**	82%	65%	
**AFC (num - bothovaries)**	44 ± 11	37 ± 7	**0.0001**

*BMI* body mass index; *HOMA-IR* homeostatic model assessment of insulin resistance; *WHR* waist to hip ratio; *T* total testosterone; *SHBG* sex hormone binding globulin; *FAI* free-androgen-index; *Δ4A* Δ4-androstenedione; *DHEAS* dehydroepiandrosterone sulfate; *F-G* Ferriman–Gallwey score; *LH* luteinizing hormone; *FSH* follicle stimulating hormone; *PCOM* polycystic ovarian morphology; *AFC* antral follicle count

**Table 3:** Multivariate logistic regression analysis of factor associated with drop-out events

Variables	OR	95%CI	***P*** value
**T (ng/mL)**	18.723	1.110–315.880	0.042*
**SHBG (nmol/L)**	0.976	0.914–1.043	0.479
**FAI %**	0.982	0.880–1.095	0.739
**DHEAS (μg/dL)**	0.999	0.995–1.002	0.428
**F-G**	1.065	0.993–1.143	0.078
**HOMA-IR**	1.097	0.922–1.304	0.296
**LH/FSH**	0.773	(0.461–1.297	0.330
**Δ4A (ng/mL)**	0.848	0.358–2.009	0.708
**Menses/6 months**	0.917	0.707–1.190	0.516

*T* total testosterone; *SHBG* sex hormone binding globulin; *FAI* free-androgen-index; *DHEAS* dehydroepiandrosterone sulfate; *F-G* Ferriman–Gallwey score; *HOMA-IR* homeostatic model assessment of insulin resistance; *LH* luteinizing hormone; *FSH* follicle stimulating hormone; *Δ4A* Δ4-androstenedione; *OR* odds ratio; *CI* confidence interval. *P*-value was calculated by multivariate logistic regression. Note: The dependent variable represents the number of drops-out at 12 months. The independent variables represent parameters recorded at baseline. *P < 0.05

## Discussion

The ANN approach made it possible to graphically show, through two semantic connectivity maps, the connections between the baseline features of a population of women with PCOS and the response to metformin treatment. Therefore, reliable predictors of response were identified with regard to the reduction in BMI and plasma androgen concentration. Both semantic connectivity maps found oligo-amenorrhea and elevated FAI as the strongest predictors, showing several variables in the background. The biomarker role of FAI in PCOS is presumably primarily related to changes in SHBG, which acts as a carrier of sex hormones and as a circulating sentinel of their production. A non-secondary role could be played by the relevance of the ovarian source of T, since metformin acts mainly on ovarian androgen production. Evidence for this is the prompt response we detected of T and FAI to treatment compared with adrenal androgens (Table 1, Fig. 2). The BMI reduction map also includes hirsutism and insulin resistance. Unexpected and certainly surprising is the fact that weight reduction is not a function of baseline weight. Regarding the reduction of FAI, the SCM delineates with oligo-amenorrhea, greater FAI, elevated triglycerides levels and body weight, the clinical picture of a severe metabolic and reproductive impairment at m0. The reference to low levels of DHEAS, an adrenal product, still focuses attention on ovarian hyperandrogenemia. The only variables on glucose homeostasis that SCMs exhibit are insulin resistance, expressed through HOMA and in secondary position for weight reduction purposes, and low baseline glucose levels for FAI reduction; the power of the latter connection is not very strong as shown by the same distance of GLIC LOW and GLIC HIGH from the “responder” pole [= 3] (Fig. 5).

The role played by glycemic parameters as markers of response to metformin and in particular by insulin resistance, frequently a prerequisite of treatment [11, 39], was clearly reduced by the results of our study. The effects of metformin in women with PCOS have been explained by the reduction of the insulin resistance-caused hyperinsulinemia due to increased insulin sensitivity [13] and this is the foundation on which the other “insulin-sensitizing” drugs rest [14, 40]. Pau et al., through the frequently sampled IV glucose tolerance test (IVGTT), demonstrated that metformin improves both glucose-mediated glucose disposal and acute insulin response to glucose and decreases ovarian androgen production in the absence of changes in insulin sensitivity [41]. Furthermore, metformin has been shown to reduce T levels in women with PCOS within 48 h of treatment, before any significant change in insulin resistance or other metabolic effects [42]. Indeed, in vitro studies using cultured ovarian theca cells support the direct inhibitory effect of metformin on ovarian steroidogenesis through inhibition of mitochondrial Complex I [43, 44]. The results of our study agree with these data and suggest that the effects of metformin in women with PCOS are poorly dependent on the presence of insulin resistance. It is possible that what has been believed to be a secondary effect of reducing insulin resistance is instead achieved independently of it.

Obesity exacerbates both the metabolic and reproductive dysfunctions of PCOS and its reduction is an important goal of therapy, but data regarding the impact of metformin on weight loss are conflicting. Our results show that metformin is effective in reducing body fat and support the relationships between treatment success and duration [45]. The ability of metformin to reduce androgen levels in women with hyperandrogenic PCOS is already known and some authors have suggested that the effect is more pronounced in non-obese than in obese women [46]. In contrast, in a predominantly obese population, our data show that the reduction in FAI is directly related to pretreatment BMI.

Metformin has been shown to improve menstrual pattern, hirsutism, body fat distribution and glyco-lipid profile and to have both short- and long-term positive effects. Our study experienced 9 spontaneous pregnancies (8.3% of the population). Although pregnancy was not an outcome of the study, it indirectly demonstrates recovery of ovulatory function following treatment with metformin.

We failed to demonstrate a predictive role of ovarian morphology and AFC in the response to metformin, with the exception of women who discontinued treatment, where AFC was significantly lower than those who did not. On the other hand, the involvement of PCOM among the diagnostic criteria of PCOS [3], due to its wide prevalence and the optional coexistence of the three characteristics, has led to a significant increase in the diagnosis of PCOS [47].

This study has some limitations. First, the dichotomy of HIGH and LOW values strictly depends on the median values of the population examined. Larger studies are needed to develop generalizable cut-off values. Another limitation is the high dropout rate, although it may provide an opportunity for some considerations.

Inconsistency and unpredictability of response to metformin are the main reasons why it is not widely used. In our study, 12.9% of women discontinued treatment because of unbearable side effects and 29.6% discontinued because they perceived it to be ineffective or because they missed follow-up. The remarkable drop-out rate of metformin treatment is not new; Ladson et al. in a 6-month randomized clinical trial reported an overall drop-out rate of 60% in the metformin arm [16]. To account for the difference in treatment efficacy, since the glycemic response to metformin is heritable, a role of polymorphisms in genes involved in the metformin transport or action has often been suggested [15, 48]. Recently, this suggestion has been refuted by demonstrating, in subjects with diabetes, that variants are not associated with decreased glucose or testosterone levels, changes in body weight, or improvements in glucose-mediated glucose disposal upon metformin use [49]. Comparing women who discontinued treatment with those who completed the 12-month follow-up, we found significant differences in baseline values, with treatment-compliant women showing greater clinical and biochemical hyperandrogenism and AFC. Notably, T and A levels are significantly associated with treatment continuation – and T could serve as a sensitive marker - while DHEAS is indifferent. Rather than a failure to retain women in a clinical trial, the drop-out rate could be the result of the coexistence of different phenotypes in the same diagnosis and the efficacy of metformin could be limited to a subgroup of women who have greater endocrine dysfunction and ovarian-derived androgen excess. If this hypothesis is confirmed, the next step should be to investigate whether the phenotype identified by clinical signs and responsive to treatment agrees with an endotype. That is, whether this subgroup of patients applies distinct and characteristic pathways.

## Conclusions

In summary, our results suggest the role of hyperandrogenemia and oligo-amenorrhea as criteria for selecting women with PCOS for metformin therapy to reduce the rate of failure and withdrawal from treatment. Our findings are consistent with increased efficacy of metformin in women with severe endocrine impairment and its success correlates with treatment duration. In addition, they also pave the way for rethinking the criteria for assessing hyperandrogenism to better define the broad population included in the diagnosis of PCOS.

## Supplementary Information

**Additional file 1..**

## Data Availability

Data available on request.
