# Supplementary material for: Hyperandrogenism and menstrual imbalance are the best predictors of metformin response in PCOS patients
Source: Reprod Biol Endocrinol. 2022 Jan 4;20:6. doi: 10.1186/s12958-021-00876-0 (PMC8729102; doi:10.1186/s12958-021-00876-0)
Supplement: Supplementary file 1 — Additional file 1.. [file 12958_2021_876_MOESM1_ESM.docx]

**SUPPLEMENTARY DATA**

**Assay methods**

- Plasma glucose concentrations, serum levels of low-density lipoprotein cholesterol, high-density lipoprotein cholesterol, and triglycerides were measured by enzymatic colorimetric assay (Vitros 5600 Ortho Clinical Diagnostic, New Jersey - USA).
- Serum insulin levels were determined using a non isotopic automated immunoassay analyzer (Tosoh AIA, San Francisco - USA).
- Serum follicle-stimulating hormone, luteinizing hormone, estradiol, T, DHEAS and progesterone levels were measured using chemiluminescence immunoassay (Vitros 5600 Ortho Clinical Diagnostics, New Jersey - USA).
- Serum SHBG levels were measured using electrochemiluminescence (Immulite 2000 Xpi ORM immunoassay system, Siemens Medical Solutions-Diagnostics,New Jersey – USA).

The intra-assay and inter-assay coefficients of variation (CV) for each aforementioned biochemical or hormonal parameter were in any case respectively lower than 6 and 11%.

**Table**. Laboratory characteristics of hormonal and biochemical data

| **Plasmatic biochemical compound** | **Method** | **Laboratory tool** | **Sensitivity of the analytical method** | **Intra-assay CV%** | **Inter –assay CV%** |
| --- | --- | --- | --- | --- | --- |
| **Follicle - stimulating hormone (FSH)** | ChemiluminescenceImmuno Assay | Vitros 5600 | 0.66 IU/l | < 2.1 | < 10L |
| **Luteinizing hormone (LH)** | ChemiluminescenceImmuno Assay | Vitros 5600 | 0.215 IU/l | < 1.5 | < 11 |
| **17 β Estradiol**  **(17-β E_2_)** | ChemiluminescenceImmuno Assay | Vitros 5600 | 6.36 pg/mL | < 4.7 | < 9.3 |
| **17-OH- Progesterone**  **(17-OH P)** | RIA | Immulite 2000 Xpi TEK 2 ORM/MET 010 |  |  | <10.5 |
| **Thyroid-stimulating**  **hormone (TSH)** | ChemiluminescenceImmuno Assay | Vitros 5600 | 0.014 μUI/mL | < 2.1 | < 2.2 |
| **Prolactin** | ChemiluminescenceImmuno Assay | Vitros 5600 | 1.4 ng/dl | < 1 | < 5.6 |
| **Androstenedione** | ChemiluminescenceImmuno Assay | Liaison Diasorin | 2.4 μg/dL | < 3.7 | < 10 |
| **Dehydroepiandrosterone sulfate**  **(DHEA-S)** | ChemiluminescenceImmuno Assay | Liaison Diasorin | 0.7 μg/dL | < 5.8 | < 10 |
| **FastingInsulin** | Non Isotopic Immunoassay | Tosoh AIA | 0.5 μU/mL | < 2.3 | < 4.6 |
| **FastingGlucose** | Enzymatic Colorimetric Assay | Vitros 5600 | 20 mg/dL | < 1.2 | < 2.2 |
| **Sex hormone binding globulin (SHBG)** | ChemiluminescenceImmuno Assay | Immulite 2000 Xpi ORM/MET 012 | 1 nmol/L | < 6 | < 8 |
| **Total Testosterone** | ChemiluminescenceImmuno Assay | Vitros 5600 | 4.9 ng/dL | < 2.4 | < 7.4 |
| **Cortisol** | ChemiluminescenceImmuno Assay | Vitros 5600 |  |  |  |
| **Total Cholesterol** | Enzymatic Colorimetric Assay | Vitros 5600 |  |  |  |
